# Significance of Linked Color Imaging for Predicting the Risk of Clinical Relapse in Ulcerative Colitis

**DOI:** 10.1155/2020/3108690

**Published:** 2020-03-06

**Authors:** Shuji Kanmura, Akihito Tanaka, Kazuki Yutsudou, Kosuke Kuwazuru, Fukiko Komaki, Yuga Komaki, Hiromichi Iwaya, Shiho Arima, Fumisato Sasaki, Shiroh Tanoue, Shinichi Hashimoto, Akio Ido

**Affiliations:** Digestive and Lifestyle Diseases, Kagoshima University Graduate School of Medical and Dental Sciences, Kagoshima, Japan

## Abstract

Ulcerative colitis (UC) is a chronic inflammatory bowel disease with unknown etiology. Recently, mucosal healing has emerged as an important therapeutic endpoint in UC. Linked color imaging (LCI) is a novel endoscopic system that enhances the color differences of the gastrointestinal mucosa. Our previous study emphasized the redness and yellowness of the lesion using LCI observation, which was useful for the evaluation of histological mucosal activity in UC. In this study, we aimed to evaluate the correlation between LCI observation and clinical relapse rate in UC patients. We retrospectively analyzed UC patients who underwent total colonoscopy between August 2016 and October 2018 at our facility with Mayo endoscopic scores of 0 or 1. We assessed the correlation between orange-like color lesion (defined as LCI-scarlet color lesions) and clinical relapse rate (requiring additional treatment for UC) during the 1-year follow-up period. Fifty-eight patients (22 female, 36 male; median age at diagnosis, 47.2 (18–80) years) who underwent colonoscopy were analyzed. During the 1-year follow-up period, clinical relapse was observed in 12 patients (20.1%) among which ten patients (83.3%) had an LCI-scarlet color lesions recognized by LCI. By contrast, 29 patients (63%) had no LCI-scarlet color lesions in the clinical remission group (*n* = 46). There was a significant difference in LCI-scarlet color between the clinical relapse and remission groups, remaining significantly associated with clinical relapse. LCI findings, including an orange-like color lesion, have diagnostic implications for predicting the risk of clinical relapse in UC during the 1-year follow-up period.

## 1. Introduction

Ulcerative colitis (UC) is a chronic inflammatory bowel disease of unknown etiology. Colonoscopy and biopsy have been established as the methods of choice to diagnose UC [1]. Recently, mucosal healing (MH) has emerged as an important therapeutic endpoint in UC [2–4]. MH has been reported to reduce the rates of disease relapse, hospital admission, and surgery, as well as lower the cumulative risk of dysplasia and colorectal cancer progression [5–8]. Endoscopic MH has been defined as a Mayo endoscopic scores (MES) of 0 or 1 using conventional white light imaging (WLI) in most clinical trials [9–12]. MH is usually diagnosed and confirmed based on endoscopic observation using WLI, as reported in a recent study [13].

Linked color imaging (LCI), a color enhancement function of the LASEREO system, has been developed as a new image-enhanced endoscopy (IEE) technology. This system makes red areas appear redder and white areas appear brighter. Therefore, it is a useful tool for recognizing color differences in the mucosa, by facilitating the detection and recognition of colorectal neoplasms [14–19].

In addition, the utility of LCI in the assessment of histological intestinal inflammation in UC patients has been reported [20, 21]. In our previous study, findings of endoscopic observations by LCI-a and LCI-b, which emphasized redness and yellowness of lesion, showed a significant difference between mucosal inflammation and non-inflammation. Therefore, mixed reddish and yellowish color site (an orange-like color) using LCI observation might be useful for detecting and visualizing mucosal inflammation. In this study, we aimed to evaluate the association between endoscopic assessment using LCI observation and clinical relapse rate in UC patients.

## 2. Materials and Methods

### 2.1. Patients

We retrospectively enrolled UC patients who underwent endoscopy between August 2016 and October 2018 at Kagoshima University Hospital. All patients were diagnosed with UC using established endoscopic, histological, and clinical criteria. Endoscopic evaluation of disease inflammatory activity was performed using MES, defined as MES 0, a normal mucosa or inactive disease; MES 1, mild activity (erythema, decreased vascular pattern, and mild friability); MES 2, moderate activity (marked erythema, lack of vascular pattern, friability, and erosions); or MES 3, severe activity (spontaneous bleeding and large ulcerations). UC patients in sustained corticosteroid-free remission (≥6 months) underwent total colonoscopy, and patients with MES 0 or MES 1 were included in the study. Patients with MES 2 or 3 or a history of total colectomy, colitic cancer, or unclassified inflammatory bowel disease were excluded. Clinical remission was defined as rectal bleeding subscores of 0 (no rectal bleeding) and stool frequency subscores of either 0 (normal stool frequency for the patient) or 1 (1 or 2 more daily stools than normal) using the partial Mayo scoring system. The rate of clinical relapse was investigated during the 1-year follow-up period. The observation started 1 year from the date of colonoscopy. The clinical relapse was defined during this period as the need for intensification or modification of medication, or UC-related hospital admission, or surgery.

### 2.2. Endoscopic Procedure

Routine bowel preparation was done by Moviprep® that contained polyethylene glycol 3350, sodium sulfate, sodium chloride, potassium chloride, sodium ascorbate, and ascorbic acid. Conventional colonoscopy was performed by an EC-L600ZW endoscope with the LASEREO system that consisted of a VP-4450HD processor and an LL-4450 light source (Fujifilm Co., Tokyo, Japan) and could produce light suitable for WLI and LCI. Two experienced endoscopists evaluated the MES using WLI and LCI endoscopic findings in each case. The endoscopic factors, including marked erythema, vascular pattern, and friability, were evaluated based on both LCI and WLI observations. The orange-like color site, emphasizing redness and yellowness by LCI observation, was defined as LCI-scarlet color lesion.

### 2.3. Assessment of Clinical Outcome

The primary endpoint was to determine the association between endoscopic findings, including LCI-scarlet color lesion and risk of clinical relapse in UC patients. The secondary endpoint was to assess predictive factor among clinical parameters for the risk of clinical relapse, which included age, gender, duration of disease, extent of disease, disease type (first attack, relapsing/remission type, or chronic continuous type), and concomitant medications at the time of colonoscopy procedure. The present study was approved by the Kagoshima University Hospital Institutional Review Board and performed in accordance with the Declaration of Helsinki. Written informed consent was obtained from all patients.

### 2.4. Statistical Analyses

Results were analyzed using the Mann–Whitney *U* test or Wilcoxon signed-rank test, as appropriate. Correlation coefficients were calculated by Spearman's rank correlation analysis. Univariate analyses were performed using the *χ*^2^ test for categorical variables and multivariate analysis using a logistic regression model with the calculation of odds ratios and 95% confidence intervals (CI). Kaplan–Meier curves for the duration of clinical remission during follow-up were generated for UC patients and were compared using the 2-side log-rank test. Kappa values were calculated for the interobserver agreement between two endoscopists to validate the MES and LCI-scarlet color. All statistical analyses were conducted using the SPSS software program (version 22; SPSS Inc., Chicago, IL, USA). *p* values <0.05 were considered statistically significant.

## 3. Results

### 3.1. Patient Clinical Characteristics

**Figure 1:**
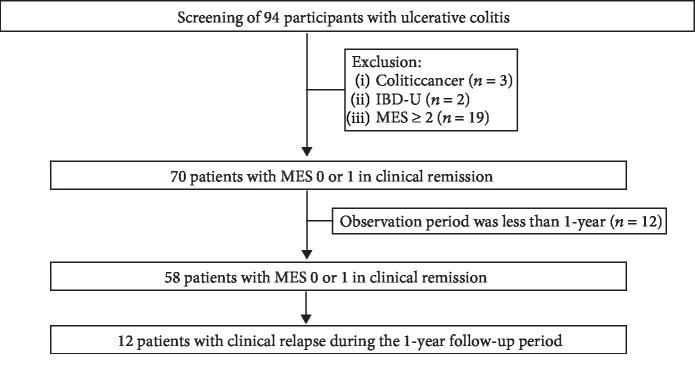
Study flowchart summarizing the selection of ulcerative colitis patients. Abbreviations: IBD-U: inflammatory bowel disease unclassified; MES: Mayo endoscopic score.

We assessed 94 UC patients who underwent endoscopy during the study period. Of these, 36 were excluded based on our exclusion criteria (colitic cancer, *n* = 3; inflammatory bowel disease unclassified, *n* = 2; MES ≥ 2, *n* = 19; endoscopic observation <1-year, *n* = 12), and 58 were analyzed (male, 36, 62%); median age, 47.2 years). During the 12-month follow-up period, clinical relapse was observed in 12 patients (20.1%; Figure 1). The median relapse time from the start of the observation period was 6.7 months (range, 2.5–11.2 months).

**Figure 2:**
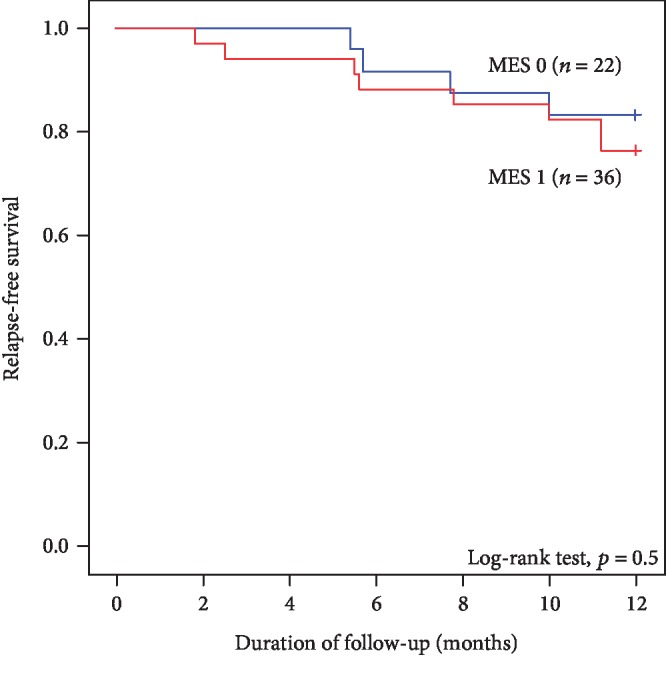
Kaplan–Meier curves of relapse-free survival for total ulcerative colitis (UC) patients with Mayo endoscopic scores 0 and 1 (*n* = 58) using the log-rank test. Clinical relapse was defined as additional or changed treatment for UC. Abbreviation: MES: Mayo endoscopic score.

For endoscopic inflammatory activity, MES 0 was observed in 22 (38%), and MES 1 was observed in 36 (62%) patients. The kappa value for MES was very good (kappa = 0.70). Thirty-three patients were treated with only aminosalicylates, eight with aminosalicylates and azathioprine, six with aminosalicylates and antitumor necrosis factor alpha (TNF*α*) agents (infliximab and adalimumab), and ten with aminosalicylates, azathioprine, and TNF*α* agents. One patient had no medication.  Table 1 summarizes the baseline characteristics of patients. Among these 12 patients, intensification of the current therapy was needed in seven, corticosteroids were started in two, and an anti-TNF*α* drug in three. The rate of clinical relapse at 1-year follow-up (requiring additional treatment for UC) showed no significant difference between MES 0 and MES 1 patient groups based on WLI observation, although the rate in patients with MES 0 group tended to be lower than that in the MES 1 group (Figure 2).

### 3.2. LCI-Scarlet Color Predicting Prognosis in UC Patients with Clinical Remission

**Figure 3:**
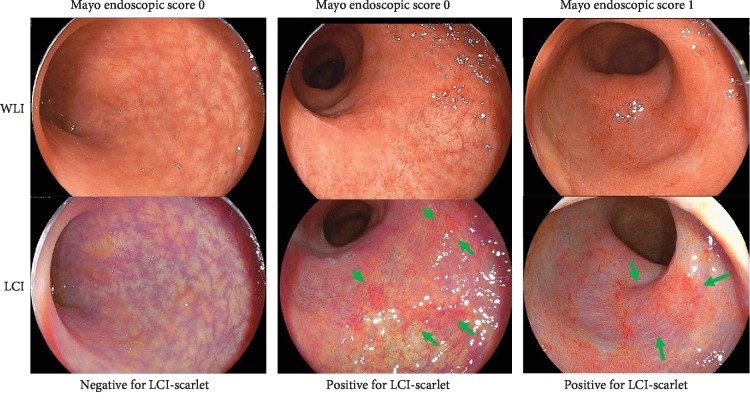
Endoscopic images of the colon segments obtained from a representative ulcerative colitis case with Mayo endoscopic score 0 and 1 observed under linked color imaging (LCI) and white light imaging (WLI). Lower panel shows the LCI-scarlet color assessment. Arrows indicate the LCI-scarlet color area.

The LCI-scarlet color was evaluated at the site that appeared to have high activity. A representative case is shown in Figure 3. The kappa value for LCI-scarlet color was very good (kappa value = 0.89).

Clinical relapse was observed in 12 patients, among whom ten (83.3%) had an LCI-scarlet color lesion recognized by LCI. By contrast, 29 patients (63%) had no LCI-scarlet color lesions in the clinical remission group (*n* = 46). Twenty-seven patients showed LCI-scarlet color lesions of which ten had clinical relapses (37.0%). By contrast, among patients with no lesions (*n* = 31), clinical relapse was observed in two patients (6.5%).

There were two cases (9.1%) positive for LCI-scarlet in MES 0 and 25 cases (69.4%) positive for LCI-scarlet in MES1. The relapse rate of UC of LCI-scarlet-positive patients in MES0 was 100% (2/2); those in MES1 was 40% (10/25).

**Figure 4:**
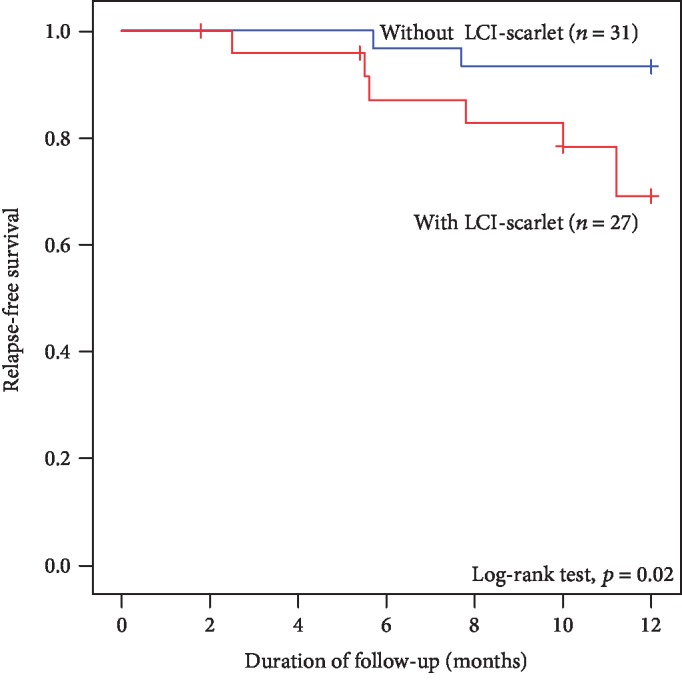
Kaplan–Meier curves of relapse-free survival for ulcerative colitis patients with or without linked color imaging (LCI)-scarlet color site using the log-rank test. Abbreviation: LCI, linked color image.

Figure 4 shows the Kaplan–Meier curves of relapse-free survival for patients based on LCI-scarlet color. The relapse rate in patients with LCI-scarlet color was significantly higher than that in patients without LCI-scarlet color (log-rank test, *p* = 0.02).

### 3.3. Correlation of LCI Findings with the Risk of Clinical Relapse in UC Patients

The duration of UC was significantly shorter in patients with clinical relapse than those with remission (average year, 7.4 vs 13.4; *p* = 0.047). Assessment of the association of LCI-scarlet color lesion with the risk of clinical relapse revealed the relapse rate of patients with LCI-scarlet color to be significantly higher than those without LCI-scarlet color (37.0% vs 3.2%; *p* = 0.002). Other clinical parameters, including gender, age, extent of disease, and current concomitant medications (5-aminosalicylic acid, azathioprine, and TNF*α*) showed no correlation with disease relapse. In multivariate analysis, LCI-scarlet color was the only independent factor that showed a significant association with clinical relapse (odds ratio, 14.8; 95 % CI, 1.68–130.4; *p* < 0.015).

## 4. Discussion

We investigated the correlation between endoscopic findings based on LCI and the risk of clinical relapse in patients with UC. The relative findings included a significant association of the scarlet color lesion under LCI observation with clinical relapse in UC patients, based on multivariable analysis. The LCI-scarlet color lesion may suggest mucosal inflammation, as reported in our previous study [21].

MH has emerged as an important therapeutic goal for UC patients. The follow-up study of the Active UC trials 1 and 2 reported that MH after 8 weeks of infliximab treatment correlated with improved clinical outcomes [8]. Although previous clinical studies defined MH as an MES of 0 or 1, it was reported that patients with MES 1 had significantly higher long-term risk of clinical relapse than those with MES 0; therefore, the concept of MH should be limited to patients with MES of 0 [8, 13, 22, 23]. A high rate of disagreement in endoscopic scoring was found in MES even among experienced physicians [24]. Although MES is the most commonly used method, its application as MES 0 or 1 in a complicated case remains controversial for the reasons mentioned above. Therefore, a more accurate and simple endoscopic observation is required. Based on our findings, we suggest the utility of LCI-scarlet color sign for the assessment of MH.

Various IEE modalities, including narrow-band imaging (NBI), blue-laser imaging, and LCI, have been developed, among which NBI and LCI are efficacious in evaluating the severity of histological inflammation in UC [20, 21, 25–27]. IEE has been frequently used in clinical practices, including detection and characterization of sporadic colonic tumor [14–19, 28–31], detection of tumor lesion, and diagnosis of histological activity, and has been recently suggested for predicting relapse risk [20, 26]. We previously reported that LCI was more useful than conventional endoscopy for the visualization and evaluation of mucosal inflammation based on the mucosal color change of redness and yellowness in UC. LCI color value correlated with histological mucosal inflammation score [21]. LCI observation emphasizes minute differences in colors, resulting in a more accurate mucosal inflammation. In the present study, LCI observations were useful for assessing the clinical outcome of UC by focusing on the color differentiation. We also reported the utility of LCI-scarlet color lesion for the prediction of clinical relapse in UC. In line with our study, Uchiyama et al. reported correlations of colonic mucosal redness enhanced by LCI with mucosal inflammation. They classified endoscopic images under LCI observation based on the degree of mucosal redness and visible vessels and suggested this LCI classification for predicting UC relapse [20]. These findings suggest that LCI would be more useful than WLI for the diagnosis of inflammatory UC.

Endoscopic evaluation of disease inflammatory activity based on MES showed no significant difference in clinical relapse rate between MES 0 and 1 patient groups; however, a trend for higher relapse rate in the patients with MES 1 was noted in this study. By contrast, the LCI finding was suggested for predicting the risk of clinical relapse in UC patients, implicating its role as a novel diagnostic approach for evaluating the colonic mucosa in UC patients for assessing MH accurately.

This study has several limitations. First, there was a single-center retrospective study design; therefore, it is necessary to consider a large-scale prospective study for assessing the diagnostic utility of LCI in UC. Second, two endoscopists evaluated the endoscopic findings using the images obtained by WLI or LCI in all cases rather than analyzing while performing the endoscopic procedure. Finally, we investigated clinical relapse based only on the requirement of additional treatment for UC as the outcome. Although we planned to consider hospitalization and surgical rates, however, due to low hospital admission rate (0.03%, 2/58 cases) and no surgical requirement (0%, 0/58 cases) during the follow-up period, we failed to assess the correlation of these rates with LCI observation.

## 5. Conclusions

For assessment of MH, LCI may be defined as no scarlet color lesion in patients with MES 0 or 1. Endoscopic findings by LCI predict the risk of clinical relapse in UC patients and are likely to aid clinicians.

## Figures and Tables

**Table 1:** Clinical characteristics of the patients with ulcerative colitis.

Number of patients	58
Gender	
Male	36
Female	22
Median age (range), year	47.2 (18–80)
Mayo endoscopic score	
0	22
1	36
Extent of disease	
Rectum	6
Left-sided colitis	13
Pancolitis	39
Current treatment	
None	1
5-ASA only	33
5-ASA and azathioprine	8
5-ASA and TNF*α* agents	6
5-ASA and azathioprine and TNF*α* agents	10

List of abbreviations: 5-ASA: 5-aminosalicylic acid; TNF*α*: antitumor necrosis factor-alpha.

## Data Availability

The data used to support the findings of this study are included within the article. Additional data are available from the corresponding author (Shuji Kanmura, skanmura@m2.kufm.kagoshima-u.ac.jp) for researchers who meet the criteria for access to confidential data.
